# Climate Change Impacts on Streamflow and Subbasin-Scale Hydrology in the Upper Colorado River Basin

**DOI:** 10.1371/journal.pone.0071297

**Published:** 2013-08-19

**Authors:** Darren L. Ficklin, Iris T. Stewart, Edwin P. Maurer

**Affiliations:** 1 Department of Geography, Indiana University, Bloomington, Indiana, United States of America; 2 Department of Environmental Studies and Sciences, Santa Clara University, Santa Clara, California, United States of America; 3 Civil Engineering Department, Santa Clara University, Santa Clara, California, United States of America; Dowling College, United States of America

## Abstract

In the Upper Colorado River Basin (UCRB), the principal source of water in the southwestern U.S., demand exceeds supply in most years, and will likely continue to rise. While General Circulation Models (GCMs) project surface temperature warming by 3.5 to 5.6°C for the area, precipitation projections are variable, with no wetter or drier consensus. We assess the impacts of projected 21^st^ century climatic changes on subbasins in the UCRB using the Soil and Water Assessment Tool, for all hydrologic components (snowmelt, evapotranspiration, surface runoff, subsurface runoff, and streamflow), and for 16 GCMs under the A2 emission scenario. Over the GCM ensemble, our simulations project median Spring streamflow declines of 36% by the end of the 21^st^ century, with increases more likely at higher elevations, and an overall range of −100 to +68%. Additionally, our results indicated Summer streamflow declines with median decreases of 46%, and an overall range of −100 to +22%. Analysis of hydrologic components indicates large spatial and temporal changes throughout the UCRB, with large snowmelt declines and temporal shifts in most hydrologic components. Warmer temperatures increase average annual evapotranspiration by ∼23%, with shifting seasonal soil moisture availability driving these increases in late Winter and early Spring. For the high-elevation water-generating regions, modest precipitation decreases result in an even greater water yield decrease with less available snowmelt. Precipitation increases with modest warming do not translate into the same magnitude of water-yield increases due to slight decreases in snowmelt and increases in evapotranspiration. For these basins, whether modest warming is associated with precipitation decreases or increases, continued rising temperatures may make drier futures. Subsequently, many subbasins are projected to turn from semi-arid to arid conditions by the 2080 s. In conclusion, water availability in the UCRB could significantly decline with adverse consequences for water supplies, agriculture, and ecosystem health.

## Introduction

The Colorado River is perhaps the most important source of water in the western United States, providing water to 30 million people, irrigating over 16,000 km^2^ of agricultural land, and producing over 8 billion kilowatt hours of hydroelectric power annually [1]. High water demand, decades of national and international treaties, and over 40 major dams render the Colorado River Basin (CRB) perhaps the most regulated watershed on Earth. With water supply and demand already in a tenuous balance in the CRB, the ability of the United States Bureau of Reclamation (USBR) as well as other state and municipal agencies to meet future water-delivery requirements and basic ecological needs in the CRB is imperiled by both climatic variability and change, as well as rising human demands on Colorado River water. Periods of drought are part of the natural climatic variability in the region. The current drought, which started in 1999 and is ongoing through the time of this writing, has exacerbated concerns by the USBR [2]–[5]. From 2000 to 2011, estimated unregulated streamflow entering Lake Powell, which is located directly upstream of Lee's Ferry, Arizona, was above average for only three years (2005, 2008, and 2011) [5]. Although a shortage of water delivered to the Lower Colorado River Basin has not been declared to date, in 2009 some local water-resources agencies were experiencing reduced water deliveries to their customers owing to precipitation declines in the previous years [6]. For example, the Metropolitan Water District of Southern California had to ration water to its customers in 2009 for the first time in nearly 20 years [6]. Further, local water-resources agencies have implemented programs to fallow land in agricultural areas, transferring the water to urban areas in need and reducing agricultural production. Regional studies for the western US, including the CRB, have documented warmer air temperatures of 1–2°C over the past several decades in the region [7]. These warmer air temperatures are connected to decreases in streamflow and shifts in snowmelt-runoff timing to earlier in the Spring, thereby depleting streamflow during the Summer season at the peak of water demands [8]–[12]. Moreover, populations in Arizona, California, and Colorado are expected to grow from 2010 to 2030 by 61, 22, and 19%, respectively, leading to a potential water-demand increase of approximately 6.2 BCM [6]. Demands for other water uses including environmental, recreational, and Native American water-rights settlements are also expected to grow.

The prospect of anthropogenic climatic changes affecting water availability in the CRB has been of great concern, and thus has been examined by many prior studies [1], [13]–[18]. While these studies are in general agreement that air temperatures will warm 2–6°C between 1990 and 2100 in the CRB, depending on the General Circulation Model (GCM) and emission scenario, the direction, magnitude, and spatial distribution of precipitation changes have been the subject of much debate. Generally, projected warmer air temperatures across the mountainous western United States, including the CRB, have been connected to a further increasing rain-to-snow ratio in precipitation, substantially decreased future snowpacks that ripen and melt earlier and drive advances in streamflow timing and runoff peaks, increased evapotranspiration (ET) during the warmer months, and decreased Summer season and annual streamflow. Precipitation changes, however, are primarily connected to the volume and seasonality of streamflow [14], [19]–[21].

Early scenario-based studies of the impacts of climatic change on water availability in the CRB suggested streamflow reductions of 30% or more [15]. More recent studies based on GCM outputs continue to suggest that the CRB is likely to become drier, albeit to a lesser degree, with mean streamflow reductions between 10–30% over the next 30–90 years [14], [16]–[18], [22]. Other studies project a declining snowpack of approximately 30% by the end of the 21^st^ century as compared to historical volumes, with extremely large declines at the lower elevations [13], [14]. Seager and Vecchi [18] found that future declines in winter snowpack may be the result of a pole-ward shift of the winter Pacific storm track. Christensen and Lettenmaier [23], who used output from an ensemble of 11 GCMs and the macro-scale Variable Infiltration Capacity (VIC) runoff model, noted modest precipitation declines of 1–2% for the A2 high greenhouse gas emission scenario. The authors found that increased evaporation from warmer temperatures had a greater effect on streamflow than precipitation changes. Very recent studies [1], [24] have cautioned against the unequivocal acceptance of the prevailing thought that the CRB will experience future streamflow-volume declines. A study by the USBR [24] found that for mountainous basins, the ensemble mean of downscaled climate projections indicate increases in Winter season precipitation. Harding et al. [1] used 112 scenarios and an ensemble of 16 GCMs to drive a macro-scale VIC hydrologic model. They found a range of potential streamflow changes from approximately −30 to 30%, where two-thirds of the ensemble runs resulted in decreases in streamflow, with the remainder showing little change or increases. The work by Harding et al. [1] illustrates that analysis results relying on few scenarios or models may be greatly influenced by model and scenario selection. Harding et al. [1] also note that the geography and topography of the CRB further complicates the understanding of hydrologic responses to future climatic changes. The northern, high-elevation, water-producing regions of the UCRB lie in a boundary region [17], [18], [22], separating an area to the north, for which precipitation increases are projected, from a region to the south, where GCM outputs forecast decreases in precipitation. These processes are complicated further by periodic depositions of dust [25], [26], which hastens snowmelt and changes basin water-volume efficiency.

Even though current state-of-the-art climate models are not able to resolve the direction and magnitude of precipitation changes for the CRB under global warming conditions, and therefore the nature of the hydrologic response with certainty, current understanding may be leveraged to determine potential futures. One goal of this study is to determine the effects of projected climatic changes on individual hydrologic components (snowmelt, groundwater, soil-water storage, surface runoff, and ET) at the smaller subbasin scale ranging from 1 to 2,000 km^2^ for the CRB. Prior studies have focused on macro-scale approaches. The future hydrologic response to climatic changes throughout the CRB is likely to differ between subregions owing to differences in elevation, soils, geology, and vegetation. As an example, subbasins at high elevations are likely to be highly dependent on snowmelt for streamflow generation [27], and subbasins with large soil-column depths are likely to be able to hold more soil-water than subbasins with smaller soil-column depths [21]. Thus, results produced at the subbasin scale can aid in understanding the difference in hydrologic response and in producing more realistic estimates of future water availability within basins. Another goal of this work, then, is to contribute to the understanding of the interaction of climate variability and change and the resultant spatially varying hydrologic fluxes contributing to water availability throughout the CRB. To this end, we assess the effects of expected climatic changes in the CRB for all hydrologic flow components at the subbasin scale by using the statistically downscaled output for an ensemble of 16 GCM models and one greenhouse gas emission scenario (A2) to drive the Soil and Water Assessment Tool (SWAT; [28]). The ensemble output enables us to assess the range of possible futures. SWAT has been successfully employed to assess water supplies in many regions of world [21], [29], [30] but has yet to be applied in the Colorado River Basin. As a large portion (∼85%) of the streamflow in the CRB is generated in the Upper Colorado River Basin (UCRB) above Lee's Ferry, Arizona [23], this study is confined to the UCRB.

## Methods

### Study area

**Figure 1:**
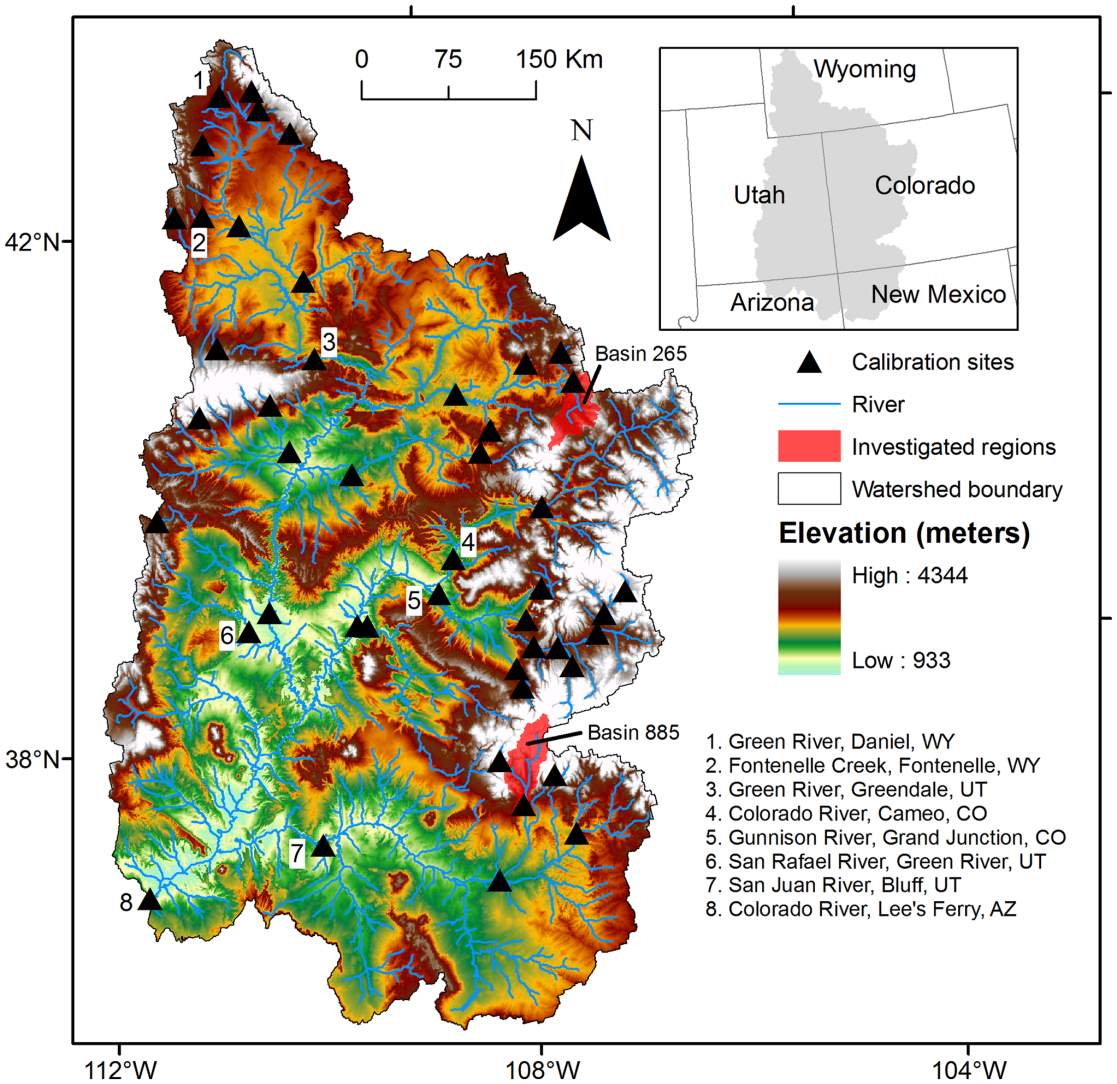
Upper Colorado River Basin study area showing calibration sites, investigated regions, and examined outlets.

The entire CRB spans much of the southwestern United States, providing water resources to seven U.S. states (Arizona, California, Colorado, Nevada, New Mexico, Utah and Wyoming) and Mexico. Climate varies considerably throughout the UCRB, varying from alpine conditions in the north and east to arid/semi-arid conditions in the south and west. 64% of the UCRB is arid or semi-arid with an average precipitation of 24 cm/year over a 190,000 km^2^ area. The Colorado River flows about 1,400 km from its headwaters in Wyoming and Colorado to the Gulf of California. Average annual temperature throughout the URCB is approximately 6°C and annual precipitation ranges from 100 cm or more in the east to less than 25 cm in the west. Precipitation in the southern portion of the UCRB is dominated by the late-Summer monsoon. High elevation snowpack in the Rocky Mountains contributes about 70% of the annual streamflow, and thus streamflow-runoff timing is dominated by Spring snowmelt [14]. Elevation ranges from approximately 4,300 meters in the northeastern portion of the UCRB to 1,200 m in the southwestern portion of the URCB near Lee's Ferry on the Colorado River (Figure 1). Land cover is dominated by rangeland and evergreen forest, which covers approximately 65 and 25% of the total UCRB land cover, respectively. The outlet for the UCRB for this study is the Lee's Ferry, Arizona gauge on the Colorado River (USGS# 09380000).

### Input data

Climatic projections from 16 GCMs and one Intergovernmental Panel on Climate Change (IPCC) greenhouse gas emission scenario (A2 (high emissions)) were used as climatic inputs to a calibrated SWAT model (Table 1). The use of multiple GCMs and scenarios allows the assessment of uncertainty around the median projection, providing more quantitative information for climate-change-impact studies [31]. Only one, intended to be the most ‘pessimistic’ high-emission A2 scenario, is considered for this study. However, present-day CO_2_ emissions exceed the IPCC A2 emission scenario, meaning that the A2 scenarios can no longer be considered “pessimistic” [32], [33], but now represent a conservative estimate of the future. All GCM output was extracted from the World Climate Research Programme's (WCRP's) Coupled Model Intercomparison Project phase 3 (CMIP3) [31] and interpolated to a 2° grid, which was then statistically downscaled using the bias-correction and spatial disaggregation (BCSD) method of Wood et al. [34], [35]. This disaggregation method has been used throughout the western United States [36]–[42]. Data include daily precipitation, maximum and minimum temperature, and wind speed from 1950 to 2099. New IPCC emissions scenarios have been developed [43], but comprehensive GCM output was not available when this research was undertaken. Early research comparing the newer Coupled Model Intercomparison Project phase 5 (CMIP5) climate models projections with CMIP3, for emissions pathways that most closely resemble A2 and B1 (specifically, the representative concentration pathways RCP 8.5 and RCP 4.5, respectively), show overall consistency between CMIP3 and CMIP5 especially with regard to warming trends, though some of the projections for reduced precipitation in the CRB may be tempered [44].

**Table 1:** General Circulation Models used in the study.

IPCC model ID	Modeling Group and Country	Reference
BCCR-BCM 2.0	Bjerknes Centre for Climate Research	Furevik et al. 2003 [64]
CGCM3.1 (T47)	Canadian Centre for Climate Modeling & Analysis	Flato and Boer 2001 [65]
CNRM-CM3	Météo-France/Centre National de Recherches Météorologiques, France	Salas-Mélia et al. 2005 [66]
CSIRO-Mk3.0	CSIRO Atmospheric Research, Australia	Gordon et al. 2002 [67]
GFDL-CM2: 2.0, 2.1	US Dept. of Commerce/NOAA/Geophysical Fluid Dynamics Laboratory, USA	Delworth et al. 2006 [68]
GISS-ER	NASA/ Goddard Institute for Space Studies, USA	Russell et al. 1999, 2000 [69], [70]
INM-CM3.0	Institute for Numerical Mathematics, Russia	Diansky and Volodin 2002 [71]
IPSL-CM4	Institut Pierre Simon Laplace, France	IPSL 2005 [72]
MIROC3.2	Center for Climate System Research (The University of Tokyo), National Institute for Environmental Studies, and Frontier Research Center for Global Change (JAMSTEC), Japan	K-1 model developers 2004 [73]
ECHO-G	Meteorological Institute of the University of Bonn, Meteorological Research Institute of KMA	Legutke and Voss 1999 [74]
ECHAM5/MPI-OM	Max Planck Institute for Meteorology, Germany	Jungclaus et al. 2006 [75]
MRI-CGCM2.3.2	Meteorological Research Institute, Japan	Yukimoto et al. 2001 [76]
CCSM3	National Center for Atmospheric Research, USA	Collins et al. 2006 [77]
PCM	National Center for Atmospheric Research, USA	Washington et al. 2000 [78]
UKMO-HadCM3	Hadley Centre for Climate Prediction and Research/Met Office, UK	Gordon et al. 2000 [79]

Input data needed for the SWAT hydrologic simulations are given in Table 2. The USBR natural flow data used for streamflow calibration are derived from climate/runoff relationships and is the streamflow that would occur if no reservoirs were present and no streamflow diversions were occurring [45]. The United States Geological Survey (USGS) Hydro-Climatic Data Network streamflow data, also used for streamflow calibration, is a streamflow and water quality dataset specifically developed for the study of surface-water conditions throughout the United States under fluctuations in the prevailing climatic conditions and hence suitable for climate-change studies [46]. Daily climate data from 1949 to 2005, including precipitation, maximum and minimum temperature, and wind speed, were obtained from gridded observed meteorological data [47].

**Table 2:** SWAT input data for historic and future scenarios for the Upper Colorado River Basin.

Description	Reference	Application	Source
30 meter Digital Elevation Model	Gesch et al., 2002 [80]	Watershed delineation and stream slopes	http://ned.usgs.gov/
National Land Cover Database	Homer, 2001 [81]	Land use properties	http://www.mrlc.gov/
State Soil Geographic Database (STATSGO)	Wolock, 1997 [82]	Soil properties	http://soildatamart.nrcs.usda.gov/
1/8 degree resolution daily climate data	Maurer et al., 2002 [47]	Precipitation, maximum and minimum temperature, wind speed input data	http://www.engr.scu.edu/~emaurer/data.shtml
Unimpaired observed streamflow data	USBR, 2005 [45]; Slack et al., 1993 [46]	SWAT model calibration	http://www.usbr.gov/lc/region/g4000/NaturalFlow/
			http://pubs.usgs.gov/wri/wri934076/1st_page.html

### Hydrologic model

SWAT is a basin-scale model designed to simulate watershed and water-quality processes, simulating the entire hydrologic cycle, including surface runoff, snowmelt, lateral soil flow, ET, infiltration, deep percolation, and groundwater return flows. For this study, surface runoff was estimated using the Soil Conservation Service Curve Number [48]. Any water that does not become surface runoff enters the soil column. Soil-water can be removed by ET, deep percolation into the deep aquifer, or move laterally in the soil column for streamflow contribution [49]. In SWAT, a deep aquifer is a confined aquifer and is assumed to contribute to streamflow outside of the watershed of interest [49]. Groundwater contribution to streamflow can be generated from shallow and deep aquifers and is based on the groundwater balance. The Penman-Monteith method was used to estimate ET [50], [51]. Relative humidity and solar radiation inputs were generated based on nearby weather gauges using the built-in SWAT stochastic weather generator. SWAT uses a temperature index-based approach to estimate snow accumulation and snowmelt processes based on the work of Fontaine et al. [52]. The model was run at a monthly time step for historic (1960-1990) and future climate scenarios (2040–2099). A technical description of SWAT can be found in Neitsch et al. [49].

This study employs simplifying assumptions. We assume constant land use/land cover throughout the 21^st^ century, as we are modeling the upper and more remote regions of the CRB only, and land use/land cover change scenarios for the UCRB were not available. We do recognize that land cover changes resulting from climatic changes and other human activity may affect the hydrology in ways that are not considered here. We also assume a constant atmospheric CO_2_ concentration within SWAT throughout all model simulations. The effect of CO_2_ on plant growth and transpiration, and thus ET, can be significant or moderate for highly vegetated watersheds [53]–[56], and therefore the decreases in streamflow and hydrologic components can be assumed to be conservative estimates and would be higher than if modeled with the effects of CO_2_. Additionally, SWAT has a simplified groundwater algorithm where groundwater contributes to streamflow only if the water stored in the shallow or deep aquifer exceeds a specified water table height [49].

### Hydrologic model calibration and validation procedure

An automated calibration technique using the program Sequential Uncertainty Fitting Version 2 (SUFI-2; [57]) was used to calibrate SWAT at 46 naturalized and unimpaired streamflow outlets within the UCRB (Figure 1). These outlets include gauges on the main stem rivers and tributaries and in many locations and elevations throughout the UCRB. Using SUFI-2, sensitive initial and default parameters relating to hydrology were varied simultaneously until an optimal solution was met. It should be noted that owing to the automatic calibration process, where each streamflow gauge is weighted equally towards a final Nash-Sutcliffe (NS; [58]) objective function, and the use of global parameters in SWAT, calibration results for individual gages may be less optimal as compared to calibrations for individual watersheds.

Three model-efficiency statistics were used to assess model performance: [1] the coefficient of determination (R^2^), [2] a modified efficiency criterion (*φ*; [59]), and [3] the NS coefficient. *φ* is the coefficient of determination, R^2^, multiplied by the slope of the regression line, *b*. This function accounts for the difference in the magnitude of two signals (captured by *b*) as well as their dynamics (captured by *R^2^*). For R^2^, *φ*, and NS, a perfect simulation is represented by a value of 1. A split-sample approach was used for calibration and validation with the calibration and validation years differed at each outlet depending on streamflow data availability (Table S1). A model warm-up time period of one year was used from 1949–1950. A complete description of the calibration procedure can be found in our previous Sierra Nevada SWAT paper [21].

### Data Analyses

#### Statistical Analyses

The impact of potential climate change on streamflow and hydrologic components was evaluated by comparing simulations using the GCMs in Table 2 in Ficklin et al. [21] under the A2 emission scenario for two future time periods: 2050s (2040–2069) and 2080s (2070–2099) to those of the historical time period (1960–1990). Precipitation, temperature, and streamflow projections are presented as the median, 1^st^ quartile (25^th^ percentile), or 3rd quartile (75^th^ percentile) values of the GCM ensemble. When describing the average of a time period (i.e. 2050 s), this value is the average of the medians or quartiles for that time period (average of median output for all Spring seasons, for example). *t*-tests for dependent samples were used to compare climate change and historical time periods with a target level of significance of *α = *0.05. The Pearson correlation coefficient was used to measure the correlation between hydrologic component changes during the 2050 s and 2080 s under the A2 emission scenario with a target level of significance of *α = *0.05. Spring season is defined as April, May, and June; Summer season is defined as July, August, and September. Hydrologic changes were summarized for the Spring and Summer seasons because they are important for aquatic species (spawning and migration) and for agricultural, industrial, and urban water use, as these are the seasons when reservoirs are replenished and the need for water is greatest, respectively.

#### Hydrologic Component Index

To determine the relative contribution of hydrologic components towards streamflow in a subbasin, the hydrologic component index (HCI) was developed by Ficklin et al. [21]. The HCI is the amount of annual snowmelt divided by sum of the annual surface and subsurface flows (lateral soil and groundwater flows). Thus, a value over 1 indicates that a large portion of streamflow is from snowmelt, whereas a value less than 1 indicates that most of streamflow originates from surface and subsurface flow. This index is useful in demonstrating where streamflow is being originated and is useful for determining regime shifts in the hydrology of a basin (i.e. from snow-dominated to rain-dominated) under a changing climate.

#### Aridity Index

The United Nations Educational, Scientific and Cultural Organization (UNESCO) aridity index (AI) was used to assess the degree of dryness for each subbasin. This AI is based on the ratio between annual precipitation to annual potential ET [60]. There are a number of different methods to estimate AI; the established UNESCO AI allows for comparison to other studies and allows the user to determine the moisture and vegetation regime of a basin and its evolution over time (Table 3).

**Table 3:** Description of aridity indices ranges [60].

Moisture regime	Description	Aridity Index value
**Hyper-arid**	**Drylands with few scrubs**	<0.03
**Arid**	**Pastoralism and no farming except with irrigation**	0.03–0.20
**Semi-arid**	**Can support rain-fed agriculture**	0.20–0.50
**Sub-humid**	**Can sustain agriculture for 180–280 growing days**	0.50–0.75
**Humid**	**Rain forests and derived savannahs**	>0.75

## Results and Discussion

### Hydrologic simulations

Overall, the model efficiency statistics indicate a satisfactory calibration (Table S1). The term “satisfactory” was based on the model efficiency requirements assessed by Moriasi et al. [61], where a calibration with NS value >0.5 (with other efficiency statistics used for confirmation) was considered to be a satisfactory calibration. The average NS coefficient for the calibration and validation periods was 0.71 and 0.72, respectively, with a standard deviation of 0.1 for both time periods. The lowest NS values were found in the tributaries and headwaters within the CRB. The average of R^2^ for the calibration and validation time periods was 0.79 with a standard deviation of 0.1. Average *φ* for the calibration and validation time period was 0.67 and 0.69, respectively, indicating that the SWAT model adequately captured the natural monthly streamflow variability.

For further validation, we calculated the differences between streamflow derived from the GCM-driven median historical simulations (1960–1990) and observed streamflow values for the historical time period (1960–1990) at four major outlets in the URCB (Colorado River at Lee's Ferry, Arizona, San Juan River near Bluff, Utah, Colorado River near Cameo, CO, and Green River at Greendale, Utah) (Figure 1). Our calculations showed that the differences between observed and GCM-driven historic simulated climate were negligible, with an average annual percent difference of less than 10%.

### GCM projections

**Figure 2:**
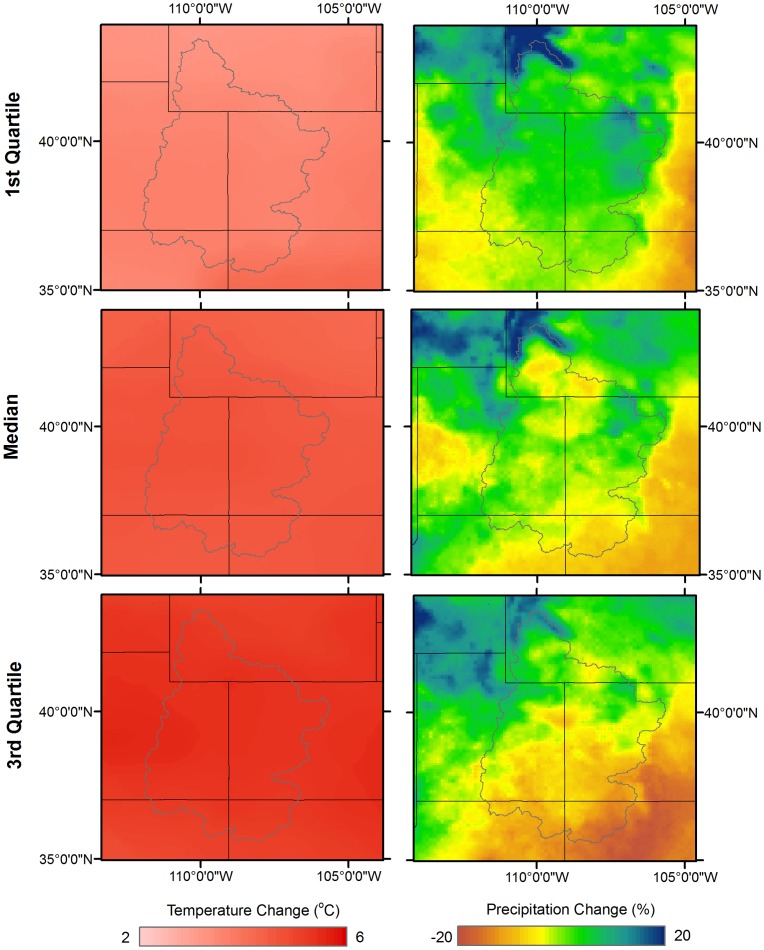
Quartiles of the projected annual temperature and precipitation change for the Upper Colorado River Basin region. The gray polygon indicates the location of the Upper Colorado River Basin study area.

Downscaled output of the GCM ensemble for the CRB indicated that air temperature is highly likely to increase under the A2 emission scenario (Figure 2), with median projections that suggest a spatially uniform average UCRB warming of 4.7°C by the end of the century as compared to the 1960–1990 air temperature average. The 1^st^ and 3^rd^ quartiles of the GCM projections indicated an overall warming of the UCRB, with a temperature increase of 3.7 and 5.4°C, respectively. These projections were in agreement with prior studies, which point to average temperature increases from 1.2 to 6.2°C by 2100 for a wider range of emissions scenarios than considered here [1], [18], [23].

Downscaled projections of precipitation, however, varied substantially among GCMs with variations in both precipitation volume and spatial patterns (Figure 2). The median of the GCM projections indicated small (<±5%) increases and decreases in precipitation throughout the UCRB with increases mostly at higher elevations and an overall average decrease in precipitation of approximately 5% compared to historical volumes. Extreme decreases (>15%) in precipitation were projected for many lower and southern portions of the UCRB for the 1^st^ quartile of GCMs, with an UCRB average decrease of 15% (Figure 2). Conversely, precipitation increases throughout the UCRB were found for the 3^rd^ quartile projections, with an average increase of 12% for the A2 emission scenario. The largest increases (15–25 %) were projected for the northernmost portion of the UCRB (Figure 2), while for the southern two-thirds of the UCRB precipitation increases of up to 10% were found. The magnitude and range of projected precipitation changes were in agreement with other recent CRB studies [1], [18], [23].

### Future hydrology

#### Changes in streamflow volume and timing

The GCM-projected changes in precipitation and temperature drive changes and shifts in streamflow and other hydrologic components. Average annual streamflow decreased for the A2 emission scenario at the Lee's Ferry outlet (the outlet for the entire UCRB) (Figure 1) by 19 and 23% for the 2050 s and 2080 s, respectively, based on the historical annual streamflow average of 585 m^3^/s (Table 4). Additionally, the hydrologic output from the GCM ensemble range indicated a projected decrease of 44% for the 1^st^ quartile and an increase of 15% for the 3^rd^ quartile during the 2050 s compared to the historical average at the Lee's Ferry outlet (Table 4). For the 2080 s, results indicated a 50% decrease in streamflow for the 1^st^ quartile of the GCM ensemble precipitation projections and a 15% increase for the 3^rd^ quartile (Table 4). All differences between mean historical and mean future streamflow projections at the Lee's Ferry outlet were found to be statistically significantly different at α = 0.05. These values are within the range of future projected decreases found in other CRB studies [1], [14], [23], [62], [63], and lower than those by Milly et al. [17], and Seager et al. [22].

**Table 4:** Average subbasin streamflow changes (in percent) for the Upper Colorado River Basin.

	Annual*		Spring		Summer	
	2050s	2080s	2050s	2080s	2050s	2080s
1st Quartile	−44	−50	−61	−66	−69	−75
Median	−19	−23	−36	−44	−46	−55
3rd Quartile	15	15	2	−13	−5	−14

* Percent change at Lee's Ferry, the outlet of the Upper Colorado River Basin.

**Figure 3:**
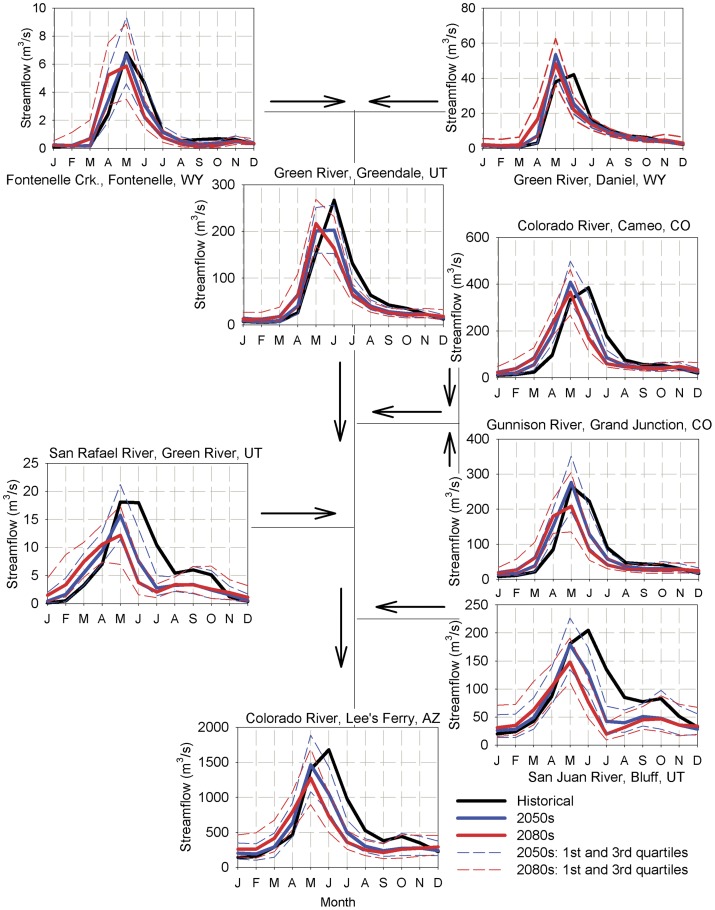
Median and quartile hydrographs of the selected outlets shown in Figure 1.

In addition to changes in streamflow volume, the SWAT model projected systematic advances in the timing of runoff through the end of the century. Figure 3 displays the average hydrographs and propagation of streamflow changes from the headwaters to the Lee's Ferry outlet (see Figure 1 for outlet locations) for the 2050 s and 2080 s. For the northern UCRB, model results suggested a streamflow timing shift of up to 1–2 months earlier for the 2050 s and 2080 s with concurrent increases in annual flow volume by approximately 25%. As the median projected precipitation decreases in this region were generally less than in the southern UCRB (Figure 2), the changes in hydrology in the northern UCRB are likely predominantly connected to increased temperatures, and manifest as shifted snowmelt timing, rather than changes in precipitation. Results for the southern UCRB pointed to decreases in future annual streamflow volume (∼15% decrease in streamflow ensemble median) driven by the projected decreases in precipitation and the regional warming shown in Figure 2. The results indicated by the quartile ranges in Figure 3 signified large variations in possible hydrologic futures based on the differences in GCM projections, with decreased average monthly flows by 40% for the 1^st^ quartile relative to the mean and increased average monthly flows by 95% for the 3^rd^ quartile (mainly from increased precipitation and large peak streamflow runoff shifts into the Winter and Spring) for the northern UCRB. In the southern UCRB, results implied a 44% decrease for the 1^st^ quartile and 81% increase for the 3^rd^ quartile. Thus, while the median of model results indicated an overall decrease in streamflow volume for both future time periods, GCM ensemble members exist that suggest scenarios of large streamflow increases for the UCRB. These projected streamflow increases for the 3^rd^ quartile were largely due to increased precipitation (∼20–25% compared to historical averages) and shifted streamflow timing towards earlier in the year for hydrologic components as discussed below.

**Figure 4:**
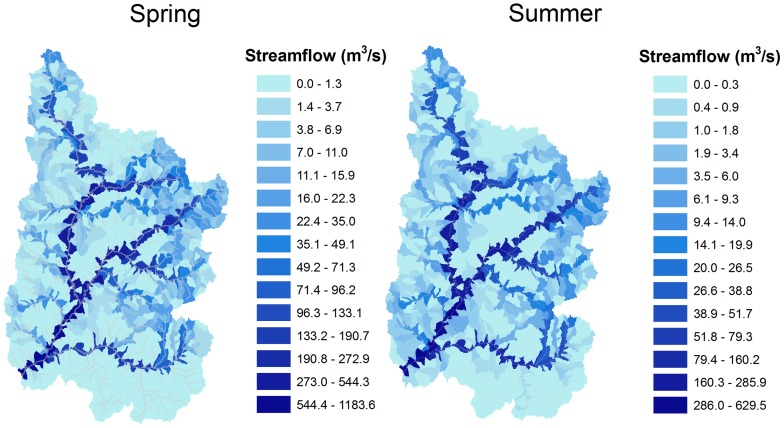
Subbasin historical streamflows of the Upper Colorado River Basin.

**Figure 5:**
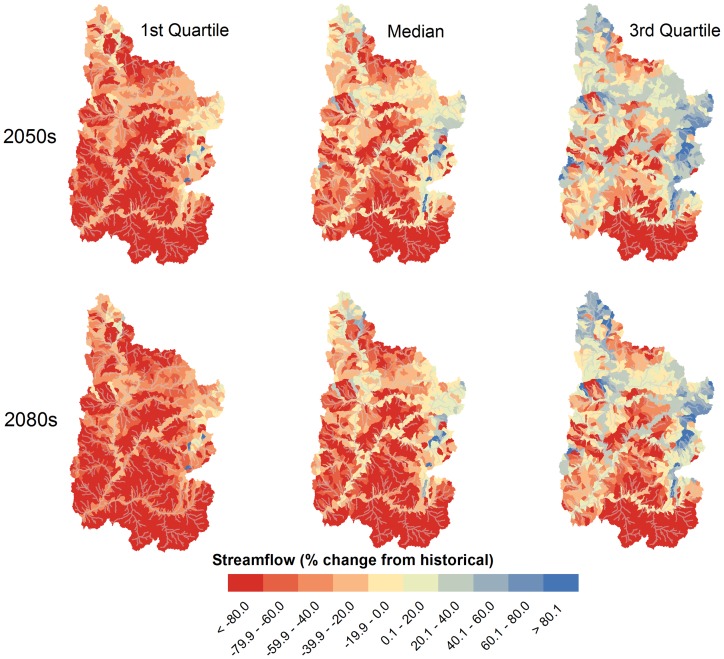
Spring median and quartile subbasin streamflow changes for the 2050 s and 2080 s for the Upper Colorado River Basin under the A2 emission scenario.

**Figure 6:**
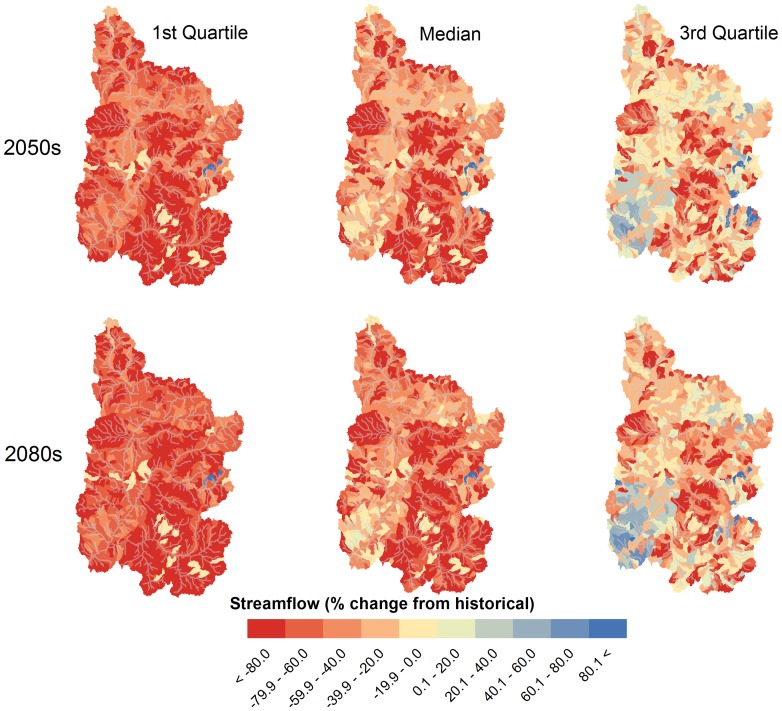
Summer median and quartile subbasin streamflow changes for the 2050 s and 2080 s for the Upper Colorado River Basin under the A2 emission scenario.

The shifts in seasonality projected for the major outlets shown in Figure 3 exhibited significant spatial variability when considered at the subbasin scale. Consistent with the expected future warmer temperatures and the advanced streamflow timing shown in Figure 3, the Winter season (not shown) median projected average subbasin streamflow decreased during the 2050 s and 2080 s by 1–2%, with decreased 1^st^ and 3^rd^ quartile streamflow of <1 and 3–6%, respectively. For the critical Spring and Summer seasons, the historical flows are displayed in Figure 4, while the median, 1^st^ and 3^rd^ quartile changes are shown in Figures 5 and 6. Historically, natural streamflow has been approximately twice as large in Spring as compared to Summer, with flows of about 1,200 and 600 m^3^/s, respectively, at Lee's Ferry. High flows in the main channels were in contrast to very low flows in surrounding subbasins. For the Spring season, projected streamflow declined for many of the southern and western UCRB subbasins, with some increased streamflow likely in the highest eastern and some northern portions of the watershed (Figure 5). According the model results for the Spring season, large declines are expected for the 2050 s and 2080 s, especially for the median and 1^st^ quartile projections (Table 4). For the 3^rd^ quartile, projected Spring streamflow increased by 2% during the 2050 s and declined by 13% during the 2080s (Table 4).

For the Summer season, streamflow is projected to decline significantly from historical levels for all but 10 of the 1,152 subbasins during the 2080s, as suggested by the median of the simulations (Figure 6). Simulations projected large declines in Summer streamflow for the median and 1^st^ quartile, and a moderate decline for the 3^rd^ quartile (Table 4). Summer flows in 22% of the URCB subbasins were projected to decrease by more than 90% during the 2080 s under the GCM median projections. The greatest declines are expected in the southeastern portion of the UCRB and some of the western and northern headwaters. The smallest decreases, taking place in the southwest portion of the UCRB, were due to a nominal change of Summer contributions of groundwater and lateral soil flow (see below). For the Summer 1^st^ quartile, the projected decline was statistically significant throughout the study area, while for the Summer 3^rd^ quartile, some projected increases were found in the southwestern and eastern portions of the UCRB. It is important to note that historically extremely low flows in the small tributaries have been prevalent during the Summer months, thus large decreases in summer streamflow may only represent a small volume of water in most cases. While this volume of water may not necessarily be important in terms of water resource supplies, it is likely to play a significant role in maintaining ecosystem health for small tributaries in the UCRB.

#### Spatial and seasonal changes in hydrologic components

**Figure 7:**
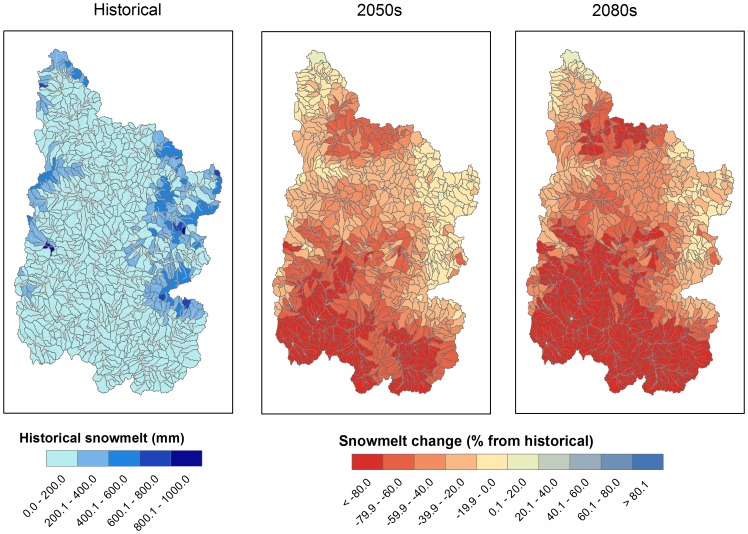
Subbasin snowmelt changes for the 2050 s and 2080 s for the Upper Colorado River Basin under the A2 emission scenario.

Analyzing the spatial distribution of individual hydrologic components and their changes indicated distinct hydrologic regimes within the UCRB. Subbasin average snowmelt decreases of 62 and 71% were projected for the 2050 s and 2080 s relative to the historical time period for the median GCM projections, respectively. Historically, the northern regions of the UCRB have been more heavily reliant on snowmelt for streamflow generation than is the southern and central UCRB (Figures 7). Streamflow changes throughout the UCRB were significantly (*p<0.05*) correlated to changes in snowmelt with a Pearson correlation coefficient of 0.31 for the 2050 s and 0.54 for the 2080 s. As shown in Figure 7, decreased snowmelt with warmer temperatures are expected to take place throughout the entire UCRB, with average declines of 36% for the 2050 s and 50% for the 2080 s for the higher elevations (>2,000 meters) and 70% for the 2050s and 80% for the 2080 s for the lower elevation subbasins (<2,000 meters). There was a significant (*p<0.*05) correlation of 0.55 and 0.61 between elevation and snowmelt change for the 2050 s and 2080 s, respectively. Snowmelt increased in 8 high-elevation northern subbasins during the 2050 s and 4 high-elevation northern subbasins during the 2080 s owing to increases in precipitation.

**Figure 8:**
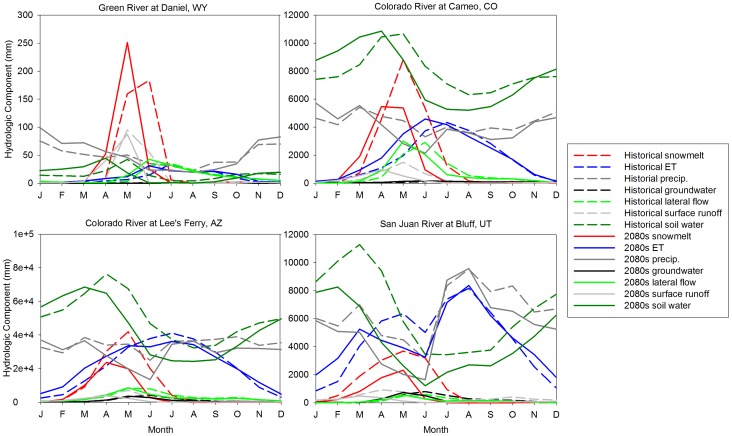
Total hydrologic component runoff values for the selected watersheds in the Upper Colorado River basin for the 2080 s under the A2 emission scenario.

The streamflow and snowmelt changes discussed above exhibited significant seasonality and reflect changes in other hydrologic flow components, as shown in Figure 8. Figure 8 displays the average monthly changes in individual hydrologic components by watershed area above four selected gauges by the end of the 21^st^ century for the median GCM projections, and indicates some general patterns. For all regions, snowmelt is expected to move from a peak in May through July to 1–2 months earlier with diminished volumes, while precipitation is projected to be slightly higher or near historical high levels in Winter, and significantly lower in April through July. The largest projected change for all outlets occurred in the timing of soil-water storage with a shift in the peak soil-water storage from May to April, resulting from early snowmelt infiltrating into the soil earlier in the year, leaving less soil water during the April through July period when temperatures and water demands on vegetation are highest. Historically, soil-water storage in all regions of the UCRB, except for the headwaters, has been a major portion of the hydrologic cycle (Figure 8). For example, the Colorado River at Cameo, CO watershed, annual soil-water storage was projected to not significantly change (*p>0.05*) with climate change, with only a 3% decrease during the 2080 s; however, the timing shift was evident (Figure 8). For the San Juan River in the southern UCRB, GCM median precipitation was projected to decrease in Fall and early Winter. In addition to a minor forward shift in soil-water storage, simulations for the San Juan River watershed showed a large decrease of soil-water storage throughout the year, largely owing to decreased precipitation and snowmelt, as well as shifts in ET. Overall, annual soil-water storage in this basin is expected to decline by 35% during the 2080 s, with the largest decreases in the late Spring/early Summer. Historically, groundwater, lateral soil flow, and surface flow have been relatively small hydrologic components for most of the subbasins in the UCRB, which are heavily dependent on the amount of water already within the soil column when a precipitation event occurs and therefore mirror temporal and spatial changes that are in soil-water storage (Figure 8). Their historic peaks have occurred in late Spring or early Summer and are projected to advance to earlier in the year by about a month by the end of the century. The northern UCRB outlets were found to be more dependent on lateral soil flow for streamflow generation.

Consistent with warmer air temperatures, future annual ET is projected to increase throughout the UCRB by, on average, 23% by the 2080 s for the median GCM projections. In addition, changes in the seasonality projected by the SWAT model simulations were consistent with the expected climatic and hydrologic shifts. Results for the northern high-elevation UCRB outlets (Green River at Daniel, WY and the Colorado River at Cameo, CO) suggested advancement of future ET by one month, leading to increased ET during the Spring months, when temperatures will have warmed sufficiently. For these watersheds, ET for the Winter months is expected to remain extremely low under projected temperatures. The San Juan River watershed in the southern UCRB historically has experienced a bi-modal temporal distribution of ET due to the Summer monsoon rains and generally warmer temperatures. Our results for the median of ensemble GCM projections suggested that this bi-modal distribution would likely become more pronounced by the end of the century. In both the San Juan River and the Colorado River at Lee's Ferry, the largest changes were projected to occur during the late Spring/early Summer with a large decrease in ET by approximately 30%. This decrease could be attributed to the significant expected decrease in precipitation during this season (Figure 8), leaving less water on the surface and in the soil-water column to be evaporated. Further, decreased snowmelt and soil-water during Spring and Summer will lead to decreased late Spring and Summer ET in this basin, in spite of higher air temperatures.

**Figure 9:**
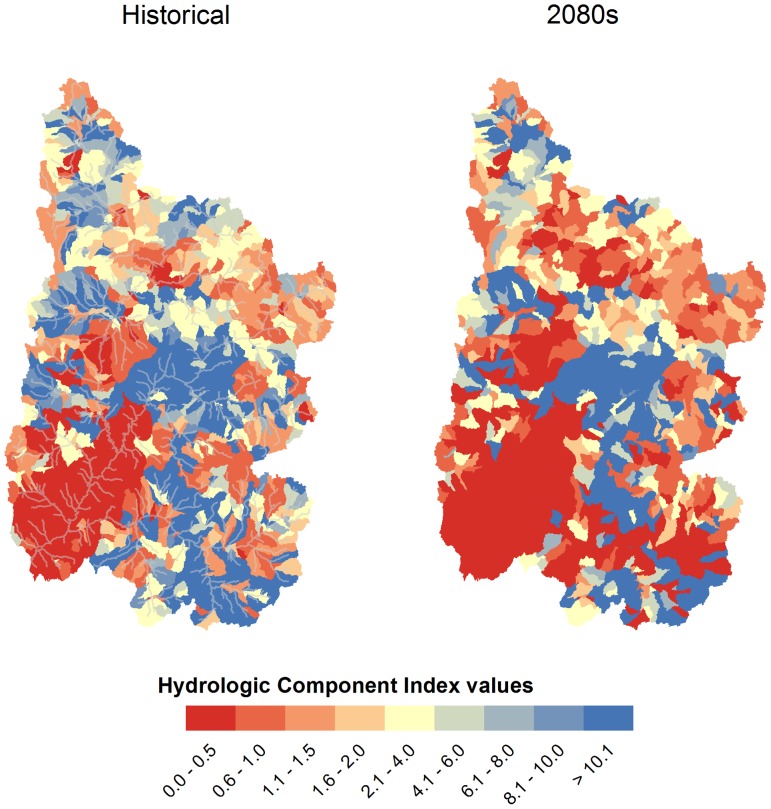
Changes in hydrologic component indices (HCI) for the 2050 s and 2080 s in the Upper Colorado River Basin under the A2 emission scenario. A value over (under) 1 indicates that a large portion of streamflow is from snowmelt (surface and subsurface flow).

Projected changes in the generation of streamflow are displayed in Figure 9. Historically, the UCRB has been dominated by areas where snowmelt contributes to a large portion of streamflow, indicated by an average HCI value of 14.1 (Figure 9). With projected climatic changes, HCI values decreased to 7.8 by the 2050 s (not shown) and to 1.8 by the 2080 s. In some regions, the projected results show a clear shift from a dominance of snowmelt-derived flows to surface and subsurface flow for maintaining streamflow volume (Figure 9). Therefore, any future changes in surface and subsurface hydrology in these regions, such as groundwater pumping for irrigation, are likely to decrease streamflow volumes significantly.

**Figure 10:**
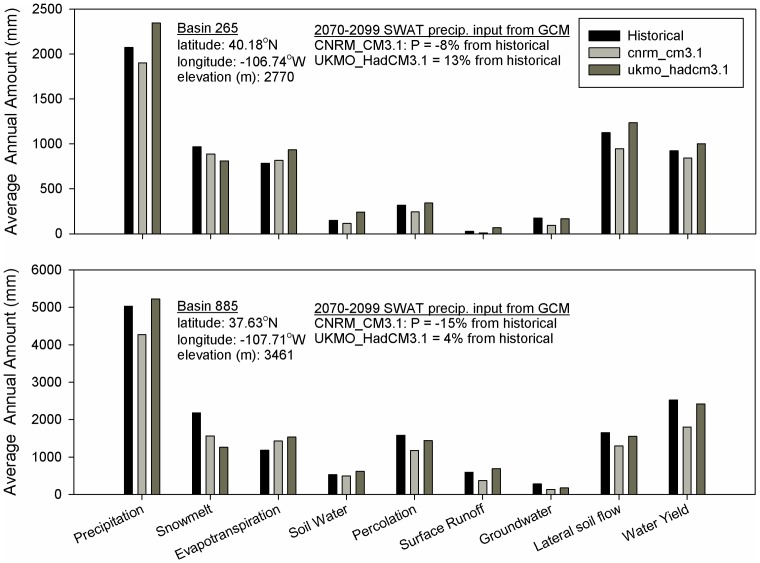
Analysis of average annual hydrologic components for two high water-generating regions and two GCM projections in the Upper Colorado River Basin. Air temperature increases for both GCM projections were ∼4.7°C.

Many studies “lump” streamflow projections, either from multiple basins or GCMs, into a single value and therefore cannot attempt to assess or explain the potential differences in water yield under either modest precipitation increases or decreases for those comparatively small areas of the UCRB that historically have driven the amount of water available in the Colorado River. As most of the water yield is generated in the headwaters of the UCRB, sensitivity to precipitation for two smaller regions important for water generation with similar temperature projections were examined. These two regions (indicated as basin 265 and 885 (region watershed outlets) in this analysis) produce large annual runoff volumes and are at different locations and elevations within the UCRB (locations and elevations shown on Figure 1). The lower elevation basin 265 (elevation of 2,770 m) historically generated 1.5% of the total average annual runoff in the UCRB, while the higher elevation basin 885 (elevation of 3,461 m) produced 3.3%. Simulation results for two GCMs, reflecting the end members in precipitation projections for the two runoff-generating basins were analyzed: CNRM_CM3.1 and UKMO_HadCM3.1 (Table 1). Both GCMs have projected a 4.7–4.8°C temperature increase for each basin at the end of the 21^st^ century, with increased precipitation of 13 and 4% (basins 265 and 885, respectively) relative to historical averages for the ‘wet’ UKMO_HadCM3.1 model (see Figure 10) and decreased precipitation of 8 and 15% for the ‘dry’ CNRM_CM3.1.

Our SWAT simulations suggest that water generation in lower basin 265 has historically been driven by average annual snowmelt and lateral soil flow, the groundwater component has been small (and thus large percent changes), and the surface runoff component negligible (Figure 10). With a decrease in precipitation by 8% as projected by the CNRM_CM3.1 model in basin 265, all hydrologic components, except ET, decreased. The total projected water yield (summation of surface runoff, groundwater contribution, and lateral soil flow contribution to streamflow) of basin 265 decreased by approximately 16%, driven by decreased lateral soil flow (9%) and groundwater component (48%). Projected snowmelt and lateral soil flow decreased approximately at the same amount as precipitation (∼10%). Vice versa, a precipitation increase of 13% as projected by the UKMO_HadCM3.1 model is projected to raise the total water yield (10%) through increased lateral soil flow (9%) and surface runoff (161%). While the surface runoff component was amplified, it remained a minor component (Figure 10). The increased surface runoff is largely due to increased soil-water storage (63%), resulting in less available capacity for precipitation infiltration. The projected warmer air temperatures decreased the snowmelt component (16%) and increased ET (19%) in spite of increased precipitation; whereas percolation, and groundwater flow remained near historic rates (7% increase and 6% decrease, respectively). Similarly, for basin 885, a decrease in precipitation by 15% as projected by the CNRM_CM3.1 model, led to decreased water yield by 28%, with concurrent declined surface runoff (37%), groundwater contribution (52%), and lateral soil flow (22%). Snowmelt (28%), surface runoff, soil-water content (7% decrease), and percolation (25% decrease) concurrently decreased, suggesting a dominating effect by rising air temperatures on the local hydrology. This was also evident by ET increases of 21%, even though precipitation was projected to decrease. Even with increased precipitation of 4% for basin 885, according to the UKMO_HadCM3.1 model, total water yield decreased by 4%. Groundwater contribution to streamflow exhibited a large decrease of 37%. Surface runoff increased by 17% from increased soil-water by 16% and decreased percolation by 9%. The largest water loss was from ET, which increased 30%. With decreased snowmelt by 42%, air temperature also had a dominating effect on the local hydrology for the UKMO_HadCM3.1 projection.

Thus, our simulation results suggest that under expected warming for the high water-generating basins in the UCRB, modest decreased precipitation would result in a yet greater decreased water yield and less available snowmelt that is important for water management and storage. In contrast, modest increased precipitation would not be expected to translate into the same magnitude of increased water yield with slight overall decreased snowmelt and increased ET. These findings may suggest that in the UCRB, whether modest warming is associated with modest precipitation changes in either direction, continued rising temperatures might make drier futures in the water-generating basins increasingly likely.

#### Aridity Index

**Figure 11:**
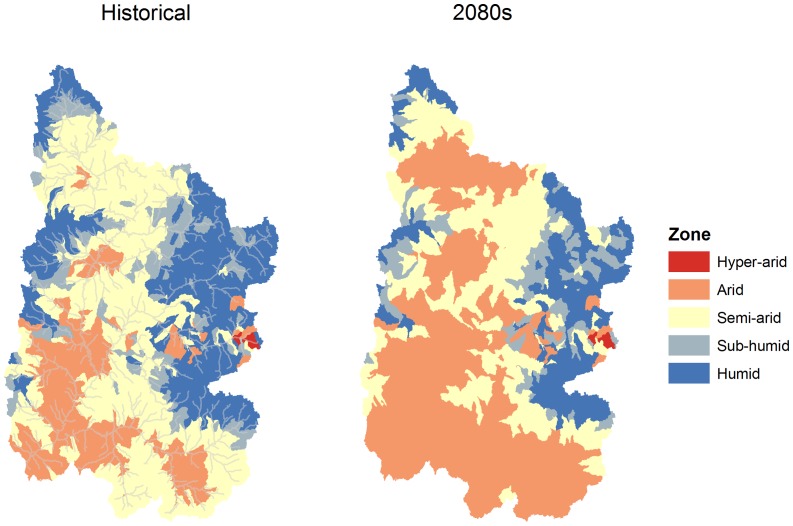
Changes in aridity indices for the 2050 s and 2080 s under the A2 emission scenario for the 2050 s and 2080 s in the Upper Colorado River Basin under the A2 emission scenario.

Based on the aridity index values calculated from our model results, the UCRB will likely become increasingly arid during the 2080 s under expected climatic changes (Figure 11). The shift from semi-arid to arid conditions is likely to be especially prominent for the southern and central subbasins of the UCRB, while the higher elevation subbasins are more likely to remain either sub-humid or humid or converted to semi-arid zones. Overall, our results did not anticipate a change in the areal coverage of hyper-arid zones within the UCRB, which historically constitutes less than 1% of the entire area (2,778 km^2^), by the end of the 21^st^ century. The largest calculated changes were found for arid zones, which increased from 316 to 637 subbasins, representing a 101% increase and a 77,400 km^2^ increase in area from the historical area of 38,500 km^2^. Concurrently, according to our simulations, the semi-arid, sub-humid, and humid zones for the GCM ensemble median could decrease by 31 (39,120 km^2^), 28 (6,816 km^2^), and 44% (31,400 km^2^), respectively, compared to the historical time period. The future transition to arid or hyper-arid conditions was also apparent for the 1^st^ and 3rd aridity indices quartiles for the 2080 s, where 62and 46% of the subbasins for the UCRB were considered arid under future climatic conditions, as compared to 25% for the historical time period.

## Conclusions

Decreased streamflow from changes in hydrologic components (surface runoff, subsurface flow, soil-water storage, and ET) at the subbasin-level owing to climate change could have significant impacts on water resources in the UCRB. A calibrated and validated SWAT model of the UCRB including 46 unimpaired streamflow sites was used to investigate the effects of climate change using 16 GCMs under the A2 emission scenario. While all models predicted surface air warming by several degrees for the area, the direction and magnitude of projected precipitation varied with GCM model, indicating modest precipitation decreases for all but the highest elevations. Median and average projected climatic changes in the UCRB suggested increased precipitation (∼5% compared to present-day volumes) at the very highest elevations, and no changes or declines (∼10%) for the middle and low elevations, with air temperatures increases of about 5°C by the end of the 21st century. Based on the different climatic driving scenarios, our simulation results indicated a range of possible hydrologic futures under all but certain warming in the area and modest precipitation changes. While we agree with the cautioning by Harding et al. [1] against a universal acceptance of large future flow decreases in the UCRB, it is important to note, that in spite of some GCMs projecting increased precipitation in critical areas of precipitation for the UCRB, current state-of-the art climate modeling suggests a somewhat drier future for the UCRB much more likely than a wetter. Our model simulations exhibited significant spatial variability in the effects of projected climate changes throughout the UCRB, which was captured by our subbasin modeling approach. For the northern UCRB, model ensemble average projections indicated a forward shift in streamflow-runoff timing with concurrent small overall reduced streamflow volume. For the southern UCRB, earlier streamflow peaks and lower streamflow volumes are expected to decline on the order of 15–40%. For the Spring season, streamflow is projected to decline for many of the southern and western subbasins, with some increased streamflow likely in the highest eastern and some northern portions of the watershed. The projected average subbasin-streamflow declined for the Spring season by 36% for the 2050 s and 44% for the 2080 s. For the Summer season, projected streamflow significantly declined throughout the UCRB, with an average decline of 46% by the 2050 s and 55% by the 2080 s.

Analysis of hydrologic components contributing to streamflow indicated large spatial and temporal changes throughout the UCRB. Snowmelt was generally found to decline throughout the UCRB, especially at the lower elevations during the 2080s. Soil-water storage for the northern UCRB was projected to shift one month in advance with little change in volume, while soil-water for the southern UCRB showed declines of approximately 35% during the 2080 s. Generally, the other hydrologic components (lateral soil flow, groundwater, surface runoff) spatially and temporally followed this trend. Annual ET increased throughout the UCRB during the 2080 s largely owing to increases in air temperature and spatial variability of soil-water storage. HCI values in some regions of the UCRB exhibited a shift from a streamflow regime dominated by snowmelt contributions to a regime dominated by surface and subsurface contributions, indicating that, in the future, streamflow in these regions is expected to become more reliant on groundwater and surface water for streamflow generation. These changes in hydrologic components indicated, that in addition to snowmelt and the volume and timing of streamflow runoff, water availability in the soils and shallow surface, and thus water availability throughout the UCRB, is likely to be affected by projected climatic changes. Additionally, our analyses of two high water-generating regions in the UCRB suggested that under expected warming for UCRB, modest decreased precipitation is likely to result in a greater decrease in water yield and less available snowmelt, while modestly increased precipitation will not likely translate into the same magnitude of water-yield increases, with slight overall decreased snowmelt and increased ET. In the UCRB, modest warming was associated with modest precipitation increases or decreases, while continued rising temperatures may make drier futures in the water-generating basins increasingly likely.

Projected changes in the aridity indices indicate a shift towards a more arid UCRB, with an increase of 77,400 km^2^ of arid land (28% of total UCRB area) with changes in climate. These results could have several implications for the UCRB:

- [1] The shift from a “rain-fed” agriculture to a zone with “no farming except with irrigation” will add further stress to a basin already stressed for water resources. Irrigation water will need to be diverted from nearby streams/rivers or from groundwater. However, these nearby streams/rivers may also be susceptible to these same issues.
- [2] The soil within an arid landscape is more exposed and susceptible to erosion loss, potentially leading to degradation of the land and the surrounding water bodies. Further, low organic matter from aridization will decrease soil stability, leading to a high potential for wind and water erosion.

This study presents one of the first climate-change hydrologic analyses performed at the subbasin scale for the UCRB, highlighting that changes in hydrology (streamflow and individual hydrologic components) from projected climate changes can vary greatly between regions in a large basin. This study provides water-resource managers and aquatic ecologists with information at the subbasin scale. If watershed planning is done at the regional scale (such as at the county- or state-level), efforts may become unproductive due to the inclusion of a large number of smaller subbasins, multiple political and governmental boundaries, and environmental differences such as stream type, land use, and soil type. Therefore, watershed management at the subbasin scale can better serve to develop solutions for local water issues.

## Supporting Information

Table S1 **SWAT model efficiency statistics for the Upper Colorado River Basin.**
(XLSX)
